# DNA Matrix Operation Based on the Mechanism of the DNAzyme Binding to Auxiliary Strands to Cleave the Substrate

**DOI:** 10.3390/biom11121797

**Published:** 2021-11-30

**Authors:** Shaoxia Xu, Yuan Liu, Shihua Zhou, Qiang Zhang, Nikola K. Kasabov

**Affiliations:** 1 Key Laboratory of Advanced Design and Intelligent Computing, Dalian University, Dalian 116622, China; shaoxia2580@163.com; 2 School of Computer Science and Technology, Dalian University of Technology, Dalian 116024, China; yliu206@mail.flit.edu.cn; 3 Knowledge Engineering and Discovery Research Institute, Auckland University of Technology, Auckland 1010, New Zealand; nkasabov@aut.ac.nz; 4 Intelligent Systems Research Center, Ulster University, Londonderry BT52 1SA, UK

**Keywords:** DNA computing, numerical computation, matrix operations, DNAzyme

## Abstract

Numerical computation is a focus of DNA computing, and matrix operations are among the most basic and frequently used operations in numerical computation. As an important computing tool, matrix operations are often used to deal with intensive computing tasks. During calculation, the speed and accuracy of matrix operations directly affect the performance of the entire computing system. Therefore, it is important to find a way to perform matrix calculations that can ensure the speed of calculations and improve the accuracy. This paper proposes a DNA matrix operation method based on the mechanism of the DNAzyme binding to auxiliary strands to cleave the substrate. In this mechanism, the DNAzyme binding substrate requires the connection of two auxiliary strands. Without any of the two auxiliary strands, the DNAzyme does not cleave the substrate. Based on this mechanism, the multiplication operation of two matrices is realized; the two types of auxiliary strands are used as elements of the two matrices, to participate in the operation, and then are combined with the DNAzyme to cut the substrate and output the result of the matrix operation. This research provides a new method of matrix operations and provides ideas for more complex computing systems.

## 1. Introduction

DNA nanotechnology has developed rapidly in recent years, and as its main material, DNA molecules have been widely used in DNA computing [1,2,3], artificial intelligence [4,5], disease diagnosis [6], and other fields [7]. The DNA molecule follows the principle of Watson–Crick complementary base pairing, which has powerful parallel computing capabilities and excellent data storage capabilities, so it is expected to perform computational operations.

In recent years, many reports have used different bio-engineered methods to achieve complicated computing systems based on DNA [8,9,10,11]. For example, Zhou et al., constructed a novel digital comparison system by combining graphene oxide with DNA, which was able to realize the comparison of two or more binary numbers [12]. In another paper, Zhou et al., constructed a logic circuit that can compute the cube root of a 10-bit binary number (within the decimal number 1000) for the first time. This research opened up a new vision for complex computing circuits and demonstrated the outstanding ability of DNA in the field of biological computing [13]. Fan et al., used DNA strand replacement technology to design a DNA-based switching circuit to implement digital computing, providing a new strategy for the development of molecular computers [14]. Dediu et al. [15] simulated the Boolean circuit using contextual hypergraph grammars techniques and further constructed the self-assembly of DNA tiles. In addition, half adder and half subtractor [16,17,18], full adder and full subtractor [19,20,21], encoder decoder [22,23], square root calculation [24], and neuron calculation model [25] have emerged. Qian and Winfree et al. [26] proposed a “seesaw door” DNA motif based on DNA strand displacement technology, and on this basis, they further constructed a four-neuron Hopfield neural network computing model that can realize “heart guessing” [27] and winner-take-all neural network model [28]; this was breakthrough research in the field of molecular intelligent computing. The calculations performed by neural networks consist of only a few operations, and matrix operations are one of them. In fact, matrix operations can be applied to many fields, such as mathematical modeling, cryptography, chemistry, communications, and computer science. In matrix operations, the main method of computing operations is DNA strand displacement [29]. The common problem with this method is leakage. Based on the importance of matrices in molecular calculations, higher requirements have been put forward for the accuracy of matrix operations. Therefore, finding a matrix construction method that can reduce leakage is a key step.

Notably, catalytic nucleic acids (DNAzymes or ribozymes) [30,31] have had prominent applications in logic calculation [32,33,34,35], micromolecular detection (virus, miRNA) [36], biosensors [37,38,39], and signal amplification [40] in recent years because of their characteristics of specific recognition and high-efficiency catalysis. The DNAzyme is an enzyme that relies on and is affected by metal ions. Under the trigger of metal ions, DNAzyme can form a stable catalytic core and catalytic substrate. This feature is often used to detect the presence of ions [41,42]. In addition, the pH value is also a condition that affects the activity of the DNAzyme [43]. Only in a certain pH value range can it exhibit activity. When the pH exceeds a certain range, the DNAzyme may be in an inactive state and does not cut the substrate. In biological systems, the activity of the DNAzyme is also affected by its own structural changes. This feature is widely used to construct molecular logic gates [44,45], cascade logic circuits [46,47], and catalytic circuits [48]. In addition, expansive regulation [49,50,51] is a special way to regulate the catalytic activity of the DNAzyme that does not need to change the structure of DNAzyme. When the sequence of the substrate and the DNAzyme recognition domain are highly mismatched, it is necessary to introduce outside regulatory molecules to help the DNAzyme cleave the substrate. Inspired by this, we applied this kind of regulation method to the E6 DNAzyme. The introduced regulation molecule is called an “auxiliary strand.” The E6 DNAzyme cuts the substrate with the assistance of the auxiliary strand. This strategy of regulating the DNAzyme activity by introducing an auxiliary strand is naturally suitable for constructing matrix operations.

In this study, we have used the auxiliary strand combined with E6 DNAzyme to form a mechanism of the DNAzyme binding to auxiliary strands to cleave the substrate so as to realize the application of matrix multiplication and weighted sum. First, the mechanism of the DNAzyme binding to a single auxiliary strand to cleave the substrate was designed that is a three-pronged complex structure composed of the E6 DNAzyme, the auxiliary strand, and the substrate. Second, on the basis of the three-pronged complex, another auxiliary strand was introduced, innovatively constructing a mechanism of the DNAzyme binding to double auxiliary strands to cleave the substrate, and thereby forming a four-pronged complex structure consisting of an E6 DNAzyme, two auxiliary strands, and a substrate. In this mechanism, E6 DNAzyme digests the substrate only when two auxiliary strands are present at the same time. Finally, relying on this mechanism, a Boolean matrix multiplication operation was constructed. The double auxiliary strands regulating the activity of E6 DNAzyme provide a new method for matrix multiplication: the two auxiliary strands can be directly used as the elements of the matrix to participate in the calculation. 2n auxiliary strands can be combined into the n^2^ four-pronged complex structure in the presence of E6 DNAzyme to cleave n^2^ substrate strands. This research aimed to construct a new matrix operation method that not only guarantees the speed of calculation but also avoids cross-interference between DNA when a large number of DNA strands participate in the calculation. The innovative contributions of this research are as follows: (1) We propose a new method of matrix operation. The matrix multiplication operation was designed by using the permutation and combination of auxiliary strands, DNAzymes, and substrates. (2) We have used the DNAzyme’s specific digestion to reduce leakage. Compared with the previous work [29], our method uses DNAzyme binding to double auxiliary strands to cleave the substrate. In this mechanism, the DNAzyme cutting the substrate requires the connection of auxiliary strands. Without any of the two auxiliary strands, the DNAzyme cannot bind to the substrate stably and cannot cut the substrate, which effectively reduces the leakage. Thus, the DNA matrix operation we designed lays the foundation for building a more complex and larger computing platform, and has considerable potential in large-scale information processing systems.

## 2. Materials and Methods

### 2.1. Materials

All DNA strands were purchased from GenScript Biotechnology Co., Ltd. (Nanjing, China). Unmodified DNA strands were purified by polyacrylamide gel electrophoresis (PAGE), DNA strands were modified with RNA bases, and fluorophores were purified by high-performance liquid chromatography (HPLC). All DNA strands were dissolved in ultrapure water as a stock solution and quantified using a Nanodrop 2000 spectrophotometer (Thermo Fisher Scientific Inc., Waltham, MA, USA). The absorption intensity was measured at λ = 260 nm. Other chemicals were of analytical grade and were used without further purification.

### 2.2. DNA Sequence Design

The sequences of all strands in the experiment are listed in Supplementary Tables S1–S3, and these strand sequences were simulated using NUPACK to avoid unexpected hybridization structures (Supplementary Figure S1) and reduce crosstalk between unrelated domains.

### 2.3. Native PAGE

Each reaction sample (30 μL, 0.6 μM) was mixed with 60% glycerol solution (5 μL) and subjected to 12% polyacrylamide gel electrophoresis in 1× TAE/Mg^2+^ buffer at a constant voltage of 90 V for 2 h. After the polyacrylamide gel electrophoresis was completed, the gel was placed in the Stains-all solution for 1 h for staining. After staining and fading, we used a Canon scanner to image the gel.

### 2.4. Kinetic Analysis of Fluorescence

Fluorescence results were obtained from real-time PCR (Agilent, Palo Alto, USA, G8830A) equipped with a 96-well fluorescent plate reader. Fluorescence measurement of all components was conducted in 1× TAE/Mg^2+^ buffer (40 mM Tris, 20 mM acetic acid, 1 mM EDTA 2Na and 12.5 mM Mg(OAc)_2_, pH 8.0) solution; the reaction volume was 30 μL, the reaction temperature was 25 °C, and the final reactant concentration was 0.3 μM. All fluorescence experiments were performed thrice to ensure reproducibility.

## 3. Results and Discussion

### 3.1. Mechanism of the DNAzyme Binding to a Single Auxiliary Strand to Cleave the Substrate

E6 DNAzyme is a kind of DNA molecule with catalytic function isolated by in vitro selection. After cloning, sequencing and catalytic activity testing of individuals in selected populations, the structure of E6 DNAzyme was finally determined. It contains two stems that are involved in substrate binding and has a central core containing 15 highly-conserved nucleotides and a stem-loop structure of variable sequence. The loop connected to the two stems that are involved in substrate binding is the catalytic core of the E6 DNAzyme. The loop is invariant, having the sequence 5′-binding stem-AGCGAT-CACCCATGT-binding stem-3′. The E6 DNAzyme whose catalytic core has changed showed no catalytic activity, suggesting that mutations within either of the two strictly-conserved unpaired regions are not tolerated [30]. In this study, we chose Mg^2+^-dependent E6 DNAzyme as the tool enzyme for our experiment.

**Figure 1:**
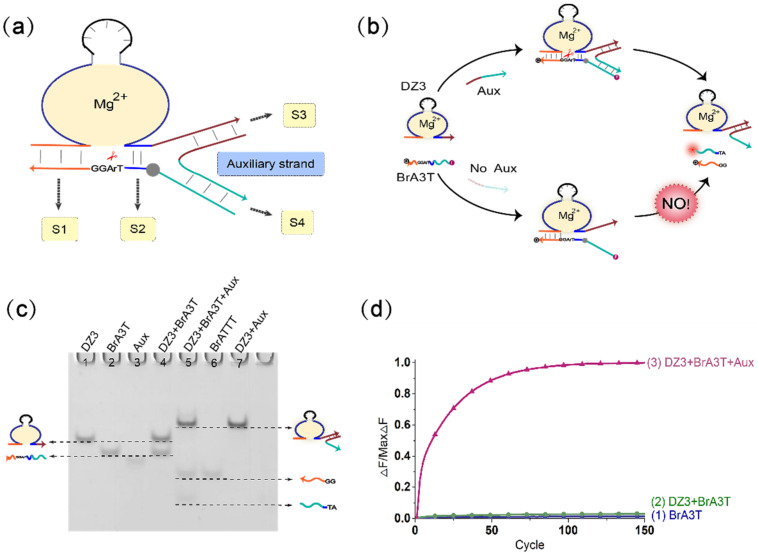
(**a**) Diagram of the mechanism of the DNAzyme binding to a single auxiliary strand to cleave the substrate. The mechanism is composed of the DNAzyme, an auxiliary strand, and a substrate strand. The three strands combine to form a three-pronged complex structure. The stems S1, S2, S3, and S4 are the important parts of the three-pronged complex structure. (**b**) The detailed process demonstration diagram of the mechanism of the DNAzyme binding to a single auxiliary strand to cleave the substrate. The 5′ end of the substrate strand is labeled with FAM fluorophore, and the 3′ end is labeled with the quencher BHQ1 for the fluorescence signal measurement. (**c**) PAGE analysis of the mechanism of the DNAzyme binding to a single auxiliary strand to cleave the substrate. The strands and complexes involved are marked above the lane number. Lane 1, DNAzyme DZ3; lane 2, substrate strand BrA3T; lane 3, input strand Aux; lane 4, DNAzyme DZ3 and substrate strand BrA3T reaction; lane 5, combination of the input strand Aux with the mixture of DNAzyme DZ3 and substrate strand BrA3T; lane 6, product strand BrATTT; lane 7, complex of DNAzyme DZ3 and auxiliary strand Aux. ([DZ3]:[BrA3T]:[Aux] = 1:1:1, [DZ3] = [BrA3T] = [Aux] = 0.6 μM). (**d**) the normalized fluorescence map of the mechanism of the DNAzyme binding to a single auxiliary strand to cleave the substrate. Curve (1), only the fluorescent substrate strand is present; curve (2), no input strand Aux is added, and only the DNAzyme DZ3 and fluorescent substrate strand BrA3T are added; curve (3), add the input strand Aux to the mixture of DNAzyme DZ3 and substrate strand BrA3T. ([DZ3]:[BrA3T]:[Aux] = 1:1:1, [DZ3] = [BrA3T] = [Aux] = 0.3 μM). The sampling interval is 4 min, 150 cycles.

The principal diagram of the mechanism of the DNAzyme binding to a single auxiliary strand to cleave the substrate is shown in Figure 1a. To optimize the DNAzyme digestion effect of the three-pronged complex structure composed of the DNAzyme auxiliary strand substrate, we verified the length of stems S2, S3, and S4. During this period, the length of stem S1 was kept unchanged (stem S1 was 7 bp). The most critical component is the length of stem S2. It is necessary to ensure that the DNAzyme can smoothly cleave the substrate when the auxiliary strand is added and to ensure that stem S2 is unstable when there is no auxiliary strand so that DNAzyme does not cleave the substrate. First, we fixed the length of stem S3 to 12 bp and stem S4 to 10 bp, and then adjusted the length of stem S2 from 0–5 bp. The result of polyacrylamide gel electrophoresis is shown in Figure S2. When the length of the stem S2 is 0 bp, 1 bp, or 2 bp, the DNAzyme cannot cleave the substrate, with or without the auxiliary strand. When the length of stem S2 is 3 bp or 4 bp, the DNAzyme starts to cleave the substrate, but only produces a small amount of cleavage products. When the length of stem S2 is 5 bp, a large amount of substrate is cleaved, but as the DNAzyme also cleaves the substrate slowly when no auxiliary strand is added (as shown in Figure S3), leakage occurs; as a result, we ruled out 5 bp as a candidate length for S2. Next, we optimized the case where stem S2 was 3 bp or 4 bp. Because the rigidity of the auxiliary strand bends the substrate so that the substrate cleavage site deviates from the catalytic core of the DNAzyme, a buffer area (base TA) was added between stem S2 and stem S4 to increase the substrate strand flexibility and achieve the best catalytic effect. As shown in Figure S4, lanes 3 and 9 have no buffer area added, whereas lanes 5 and 11 have an added buffer area. The amount of product produced by the latter is significantly greater than the amount of product produced by the former. In this mechanism, the DNAzyme cutting effect is almost the same when the length of stem S2 is 3 bp or 4 bp. Therefore, in the subsequent experiments, the length of stem S2 was selected as 3 bp. In addition, we also verified the influence of the length of stem S3 and stem S4 on this mechanism (as shown in Figures S5 and S6). The results show that when the length of stem S3 is 11 bp or 12 bp, the catalytic activity of DNAzyme is higher. When the length of the stem S4 is 6–10 bp, the catalytic activity of the DNAzyme gradually decreases with the shortening of the stem length. By 7 bp, the DNAzyme no longer functions properly; that is, the auxiliary strand loses its auxiliary catalytic effect. In short, during the operation of the mechanism, when the length of stem S2 is 3 bp, the length of stem S3 is 11 bp, the length of stem S4 is 10 bp, and the DNAzyme can better catalyze the substrate. In addition, adding a buffer area between stem S2 and stem S4 makes the entire mechanism more stable, as the DNAzyme catalytic core can more accurately identify the substrate cleavage site, and this improves the efficiency of the mechanism of the DNAzyme binding to a single auxiliary strand to cleave the substrate. The activity of DNAzyme is also affected by pH value, and it only shows activity within a certain pH value range. As shown in Figure S7, when the pH value of the reaction environment is between 8.0 and 9.0, the DNAzyme activity reaches its peak. When the pH value is lower or higher, the activity of DNAzyme is reduced or even inactivated. Therefore, in this study, the pH value of our reactant environment is 8.0.

Figure 1b shows the mechanism of the DNAzyme binding to a single auxiliary strand to cleave the substrate. The mechanism contains DNAzyme DZ3, a substrate with RNA modification BrA3T and auxiliary strand Aux. As the 3′-end binding arm of DNAzyme DZ3 is unstable in binding to the substrate BrA3T with RNA modification, DNAzyme DZ3 and the substrate BrA3T initially coexist in the solution, and the substrate is not cleaved. When the auxiliary strand Aux is introduced, a part of the auxiliary strand Aux binds to DNAzyme DZ3, and the other part of the auxiliary strand Aux serves as the binding arm of DNAzyme DZ3. At this time, DNAzyme DZ3 and auxiliary strand Aux bind to the substrate BrA3T simultaneously to form a three-pronged complex structure so that the substrate BrA3T is cleaved; this was confirmed by native polyacrylamide gel electrophoresis experiments. As shown in lane 4 of Figure 4, without the auxiliary strand Aux, DNAzyme DZ3 does not react with the substrate BrA3T, and two corresponding separate bands can be observed. When the auxiliary strand Aux is added, DNAzyme DZ3 binds to the auxiliary strand Aux and cleaves the substrate BrA3T. Meanwhile, the substrate BrA3T disappears and forms two cut products (lane 5). In addition, real-time fluorescence analysis of the mechanism was performed, as shown in Figure 1d: when no auxiliary strand is added, the fluorescence signal does not increase significantly (Figure 1d, curve 2). When the auxiliary strand is added, the fluorescence signal increases significantly (Figure 1d, curve 3). This indicates that the mechanism of the DNAzyme binding to a single auxiliary strand to cleave the substrate is successful.

### 3.2. Mechanism of the DNAzyme Binding to Double Auxiliary Strands to Cleave the Substrate

**Figure 2:**
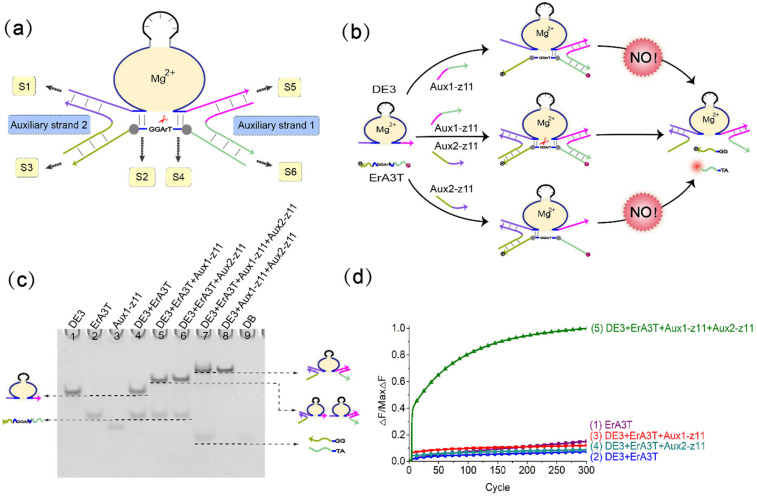
(**a**) Diagram of the mechanism of the DNAzyme binding to double auxiliary strands to cleave the substrate. This mechanism is composed of the DNAzyme, two auxiliary strands, and the substrate strand. The four strands combine to form a four-pronged complex structure. The stems S1S5, S2S4, and S3S6 are the important parts of the four-pronged complex structure. (**b**) Demonstration diagram of the detailed process of the mechanism of the DNAzyme binding to double auxiliary strands to cleave the substrate. The 5′ end of the substrate chain is labeled with FAM fluorophore, and the 3′ end is labeled with the quencher BHQ1 for the fluorescence signal measurement. (**c**) PAGE analysis was done on the mechanism of the DNAzyme binding to double auxiliary strands to cleave the substrate. The strands and complexes involved are marked above the lane number. Lane 1, DNAzyme DE3; lane 2, substrate strand ErA3T; lane 3, input strand Aux1-z11; lane 4, DNAzyme DE3 reacts with substrate strand ErA3T; lane 5, only one input strand Aux1-z11 is added to the mixture of DNAzyme DE3 and the substrate strand ErA3T; lane 6, only add another input strand Aux2-z11 to the mixture of DNAzyme DE3 and the substrate strand ErA3T; lane 7, add two input strands Aux1-z11 and Aux2-z11 to the mixture of DNAzyme DE3 and the substrate strand ErA3T; lane 8, a complex of DNAzyme DE3 and two auxiliary strands Aux1-z11 and Aux2-z11; lane 9, product strand DB. ([DE3]:[ErA3T]:[Aux1-z11]:[Aux2-z11] = 1:1:1:1, [DE3] = [ErA3T] = [Aux1-z11] = [Aux2-z11] = 0.6 μM). (**d**) the normalized fluorescence map of the mechanism of the DNAzyme binding to double auxiliary strands to cleave the substrate. Curve (1), only the fluorescent substrate strand ErA3T is present; curve (2), only DNAzyme DE3 and fluorescent substrate strand ErA3T are added; curve (3), only one input strand, Aux1-z11, is added to the mixture of DNAzyme DE3 and fluorescent substrate strand ErA3T; curve (4), only one input strand Aux2-z11 is added to the mixture of DNAzyme DE3 and fluorescent substrate strand ErA3T; curve (5), the two input strands Aux1-z11 and Aux2-z11 are added to the mixture of DNAzyme DE3 and the fluorescent substrate strand ErA3T. ([DE3]:[ErA3T]:[Aux1-z11]: [Aux2-z11] = 1:1:1:1, [DE3] = [ErA3T] = [Aux1-z11] = [Aux2-z11] = 0.3 μM). The sampling interval is 4 min, 300 cycles.

To further explore the possibility of the mechanism of the DNAzyme binding to a single auxiliary strand to cleave the substrate and increase the complexity of the system, we introduced a second auxiliary strand on the basis of single auxiliary strand mediation, and constructed a mechanism of the DNAzyme binding to double auxiliary strands to cleave the substrate, as shown in Figure 2a. Based on the above experience, we set the length of stem S2 and S4 to 3 bp, and controlled the length of stems S1, S3, S5, and S6 unchanged (wherein the length of stem S1 and S5 was 12 bp, and the length of stem S3 and S6 was 10 bp). We added a buffer area between stems S4 and S6, and between stems S2 and S3. We used native polyacrylamide gel electrophoresis to perform comparison experiments with or without the buffer area. As shown in Figure S8, lane 6 does not have the added buffer area, and lane 12 does have the added buffer area. It can be clearly seen that lane 6 has almost no digestion products, while lane 12 produces a large amount of digestion products. This shows the importance of the buffer area to this mechanism. In addition, we also verified the influence of the length of stems S1 and S5 and stems S3 and S6 on the mechanism. Keeping the length of stems S3 and S6 unchanged, we adjusted the length of stems S1 and S5 from 8 bp to 12 bp. Fluorescence analysis (Figure S9) showed that the length of stems S1 and S5 was reduced from 11 bp to 8 bp, and the cleavage activity of DNAzyme gradually decreased. The reason is that as the lengths of S1 and S5 are shortened, the binding effect of the auxiliary strands and DNAzyme gradually weakens, and the combined digestion structure tends to be unstable. In order to make the digestion effect more stable, we chose the length of the stems S1 and S5 to be 11 bp. Keep the length of stems S1 and S5 at 11 bp, and adjust the length of stems S3 and S6 from 6 bp to 10 bp. Fluorescence analysis (Figure S10) shows that when the length of stems S3 and S6 is 10 bp, DNAzyme can stably cleave the substrate. Apart from this, DNAzyme hardly cleaves the substrate. Since the result of our matrix operation is a Boolean value, we can accept it as long as DNAzyme has a significant cutting effect. The length of the stems S3 and S6 is 10 bp, which is within our acceptable range. In short, we choose the length of the stems S1 and S5 to be 11bp and the length of the stems S3 and S6 to be 10 bp, and add buffer area, the catalytic effect of DNAzyme can achieve the purpose of our Boolean matrix operation.

Figure 2b shows the process of the mechanism of the DNAzyme binding to double auxiliary strands to cleave the substrate. The mechanism includes DNAzyme DE3, the substrate ErA3T with RNA modification, and the two auxiliary strands Aux1-z11 and Aux2-z11. In this mechanism, the combination of DNAzyme DE3 and ErA3T is unstable, so DNAzyme does not cleave the substrate without adding any input. When only the auxiliary strand Aux1-z11 is added, part of the auxiliary strand Aux1-z11 hybridizes with DNAzyme DE3, and the other part hybridizes with the substrate ErA3T. However, owing to the weak binding ability, and the fact that the other part of the substrate is in a suspended state, the substrate is still free in the solution. DNAzyme DE3 does not cleave the ErA3T substrate, and no signal is generated. Therefore, when only the auxiliary strand Aux1-z11 exists, it cannot help DNAzyme DE3 perform the cleavage effect. Similarly, when only the auxiliary strand Aux2-z11 is added, DNAzyme DE3 cannot cleave the substrate ErA3T, nor does it generate a signal. However, when the auxiliary strands Aux1-z11 and Aux2-z11 are added at the same time, the binding arms at both ends of DNAzyme DE3 bind to a part of the two auxiliary strands, and the remaining parts of each of the two auxiliary strands hybridize with the substrate ErA3T, thereby forming a four-pronged complex structure of DNAzyme DE3, the auxiliary strand Aux1-z11, the auxiliary strand Aux2-z11, and the substrate ErA3T. DNAzyme DE3 cleaves the substrate ErA3T to generate an output signal.

We confirmed this mechanism through native polyacrylamide gel electrophoresis. As shown in Figure 2c, in lane 4, only DNAzyme DE3 and the substrate ErA3T are added. As seen in the figure, both have separate bands, so no reaction occurs. When only one of the auxiliary strands is added, DNAzyme DE3 binds to the auxiliary strand but does not cleave the substrate ErA3T (lanes 5, 6). When both auxiliary strands are added, the substrate ErA3T is cleaved by DNAzyme DE3 (lane 7). Real-time fluorescence analysis is also proved at this point. As shown in Figure 2d, DNAzyme does not work either when no auxiliary strand is added or when only one of them is added. When both auxiliary strands are added, the auxiliary strand helps DNAzyme DE3 cleave the substrate ErA3T and produces a strong fluorescent signal. This indicates that the mechanism of the DNAzyme binding to double auxiliary strands to cleave the substrate is successful.

### 3.3. Realization of Boolean Matrix Multiplication

**Figure 3:**
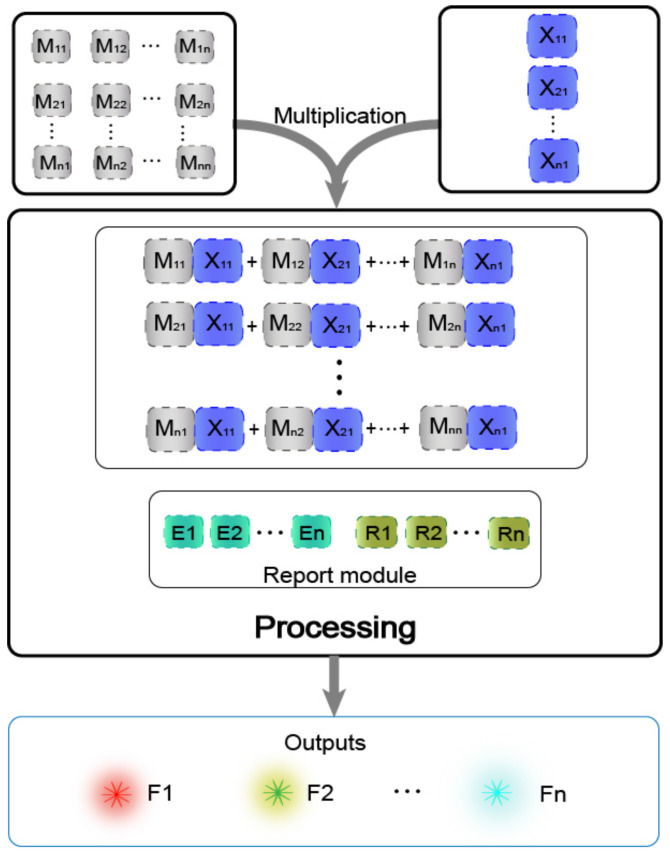
Schematic diagram of N × N matrix multiplication based on the mechanism of the DNAzyme binding to double auxiliary strands to cleave the substrate.

In previous work [29], the main method of matrix operation was the combinatorial displacement mechanism. In the absence of strands with toehold domains, a slow branch migration occurs. Therefore, we avoided the use of this mechanism and instead propose a method of DNAzyme binding to auxiliary strands to cleave the substrate. The process of calculating the value was transformed into the process of two auxiliary strands binding the DNAzyme to cleave the substrate. The DNAzyme cutting the substrate requires the binding of two auxiliary strands, and without any of the two auxiliary strands, DNAzyme does not cleave the substrate, which effectively reduces the leakage. Based on this, the mechanism is highly suitable for the calculation operation of the multiplication of two matrices. The mechanism can be applied to the calculation of the Boolean product of N × N matrices in theory. The condition that two matrices can be multiplied is that the number of columns in the first matrix must be the same as the number of rows in the second matrix. As shown in Figure 3, matrix M has N rows and N columns, making it an N × N matrix, and matrix X is a column vector with N elements, which is an N × 1 matrix, and its number of rows is the same as the number of columns of matrix M. In the mechanism of the DNAzyme binding to double auxiliary strands to cleave the substrate, one of the auxiliary strands is used as an element in matrix M, and the other auxiliary strand is used as an element in matrix X. The elements in matrix M are multiplied by the elements in matrix X, with the participation of the reporting module (DNAzyme and the functionalized substrate). The calculation result is successfully transduced to the fluorescent signal, and the calculation result is judged by reading the fluorescent signal. In the matrix, the same domain is designed between the elements of each row and the elements of each column, so that when the rows and columns of the two matrices are multiplied, they can combine different DNAzymes but can cut the same substrate to produce the same fluorescence signal.

**Figure 4:**
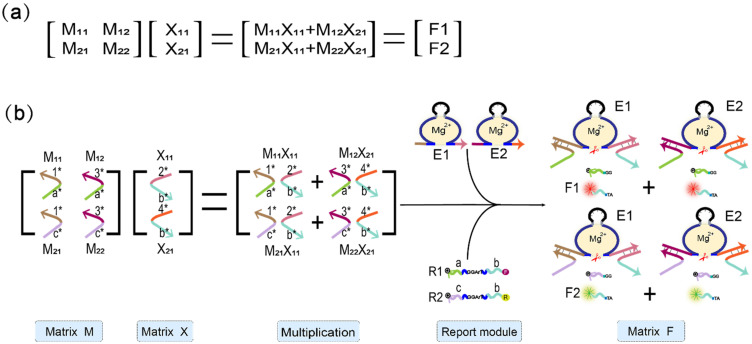
Boolean matrix multiplication reaction process diagram. (**a**) The mathematical principle of matrix M and matrix X multiplication. (**b**) Scheme diagram of the matrix M multiplied by the matrix X. The elements of the two matrices are represented by two auxiliary strands that constitute the mechanism of the DNAzyme binding to auxiliary strands to cleave the substrate. The reporting module consists of two DNAzymes and two substrate strands modified with FAM and ROX and quenching groups, respectively.

To illustrate that the mechanism of the DNAzyme binding to double auxiliary strands to cleave the substrate can be applied to matrix multiplication, we performed the multiplication calculation of 2 × 2 Boolean matrix. Figure 4a shows the mathematical principle of 2 × 2 matrix multiplication. Figure 4b is a demonstration of using DNA to realize the multiplication of two matrices. Matrix M is a 2 × 2 matrix; the first row of elements is composed of M_11_ and M_12_, the second row of elements is composed of M_21_ and M_22_, and the two elements of matrix X are X_11_, X_21_. The reporter module is composed of DNAzyme E1, E2, and two functionalized substrate strands, R1 and R2. The 5′ end of R1 modifies the fluorophore FAM, and the 3′ end modifies the quencher BHQ1. Moreover, the 5′ end of R2 modifies the fluorophore ROX, while its 3′ end modifies quencher BHQ2. First, the elements M_11_ and M_12_ of the first row of matrix M are multiplied by the two elements, X_11_ and X_21_, of matrix X, and the two combinations, M_11_X_11_ and M_12_X_21_, are obtained. Both of these combinations perform an “AND” operation; that is, if any element is missing in each combination, the calculation result of the combination is “0”. M_11_X_11_ combines with DNAzyme E1 to form the mechanism of the DNAzyme binding to auxiliary strands to cleave the substrate, which cuts the substrate R1 and generates a fluorescent signal. M_12_X_21_ binds to DNAzyme E2, which also cleaves the substrate R1 to produce the same fluorescence signal. The two combinations of M_11_X_11_ and M_12_X_21_ perform an “OR” operation; that is, when either one or both of them exist, the result is calculated as “1”; otherwise, it is “0”. Second, the elements M_21_ and M_22_ of the second row of matrix M are multiplied by the two elements, X_11_ and X_21_, of matrix X, and the two combinations obtained are M_21_X_11_ and M_22_X_21_. Similarly, the two elements of each combination perform an “AND” operation, in which both of the two elements must be present; otherwise, the combined calculation result is “0.” Combination M_21_X_11_ binds to DNAzyme E1 and cleaves the fluorescent substrate R2 to generate a fluorescent signal. The combination M_22_X_21_ binds to DNAzyme E2 and cleaves substrate R2 to generate the same fluorescent signal. The “OR” operation is performed between these two combinations. When one or both of them is present, the calculation result is “1”; otherwise, it is “0”.

**Figure 5:**
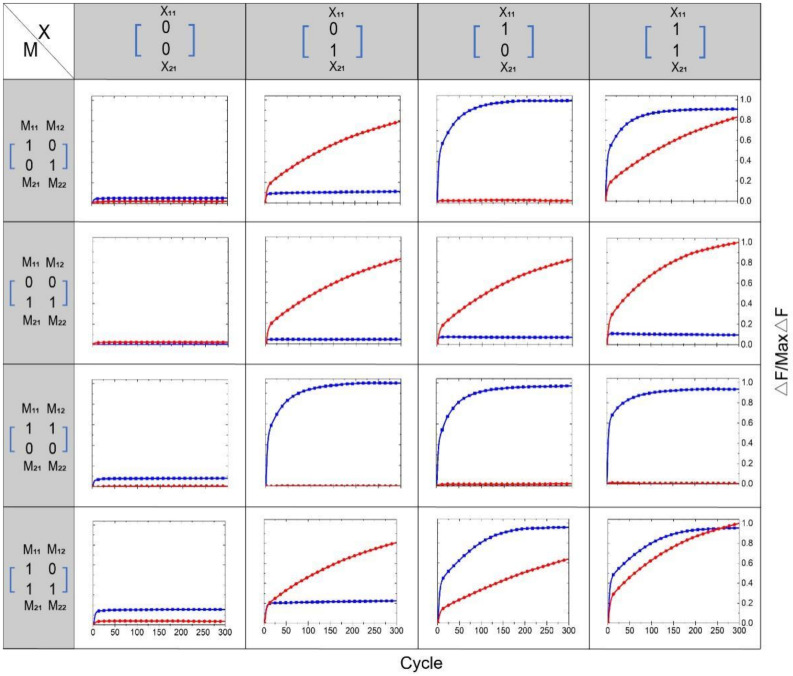
Results of 16 independent experiments. Before adding the element strands of the two matrices, the two DNAzymes and the two substrate strands of the reporting module are mixed. The sampling interval is 4 min, 300 cycles.

Regarding the multiplication of the matrix M and the matrix X, the calculation results were all represented by the output of the fluorescence signal. If the fluorescence signal rose, the result was interpreted as “1,” and if the fluorescence signal did not rise, the result was interpreted as “0.” We performed 16 groups of independent experiments; the results are shown in Figure 5. The blue curve in the figure represents the element value F1 of the first row of the output matrix F, which is represented by the fluorophore FAM. The red curve represents the element value F2 in the second row of the output matrix F, represented by the fluorophore ROX. The 16 independent experiments correspond to four different M matrices multiplied by 4 different X matrices. The matrix M has four different input strands, and the matrix X has 2 different input strands. The element values “1” and “0” of each matrix are coded by whether the input strand exists or not. The existence of the input strand represents “1,” and the absence of the input strand represents “0”. In addition, in the Supplementary Materials, we show another 16 groups of independent experiments, depicted in Figure S11. In all experiments, the element values of the first row represented by the fluorophore FAM are subjected to a unified normalization process, and the element values of the second row represented by the fluorophore ROX are subjected to a unified normalization process.

### 3.4. Weighted Sum of Boolean Matrix Multiplication

**Figure 6:**
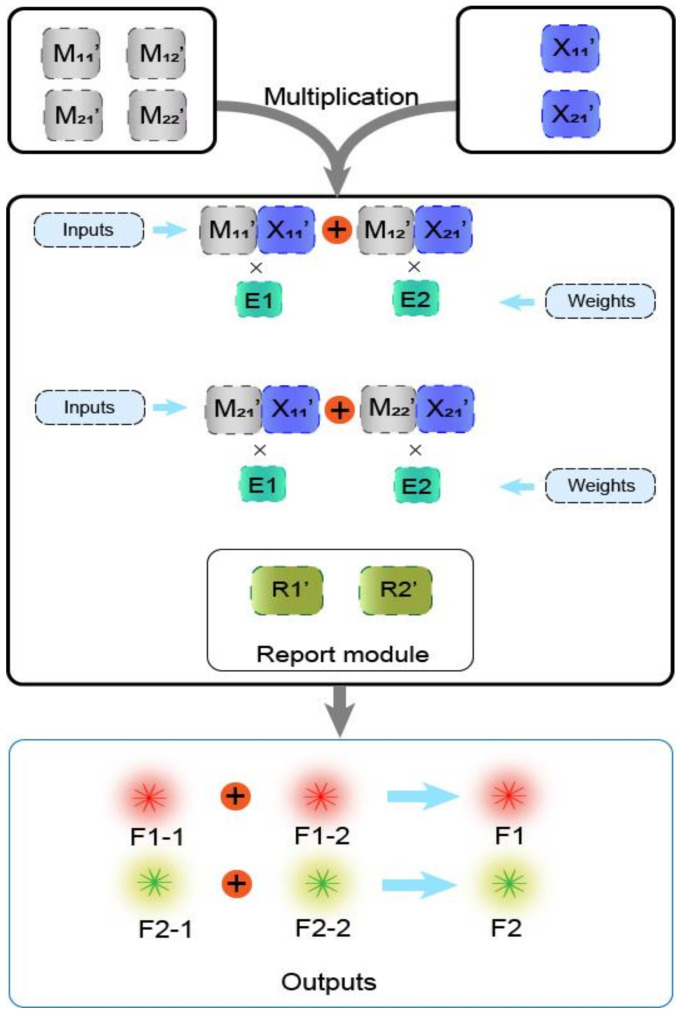
Schematic diagram of the weighted sum of 2 × 2 matrix multiplication based on the mechanism of the DNAzyme binding to double auxiliary strands to cleave the substrate.

**Figure 7:**
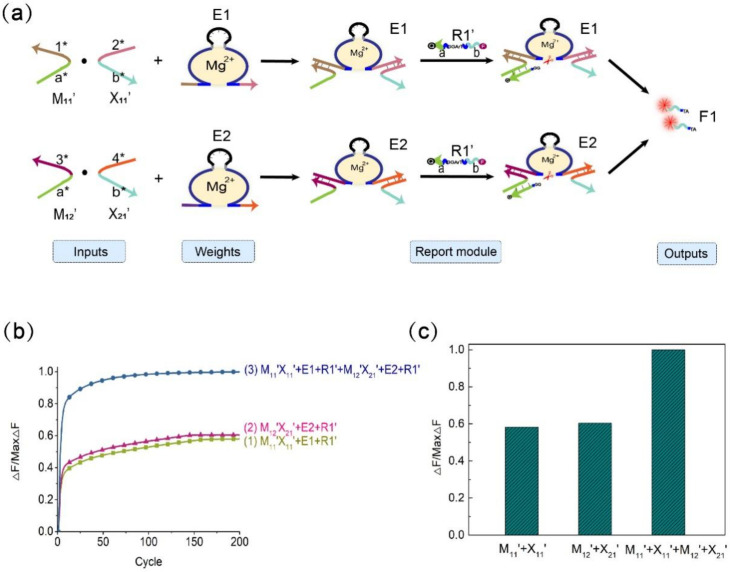
Schematic diagram of the matrix multiplication weighted sum. (**a**) Schematic diagram of the weighted sum process of the first-row value F1 of the output matrix F. (**b**) Normalized fluorescence image of F1. Curve (1), combination of M_11′_X_11′_ and weight E1, substrate R1′, [M_11′_]:[X_11′_]: [E1]: [R1′] = 1:1:1:2; curve (2), combination of M_12′_X_21′_ and weight E2, substrate R1′, [M_12′_]:[X_21′_]: [E2]: [R1′] = 1:1:1:2; curve (3), combinations M_11′_X_11′_, M_12′_X_21′_, weights E1, E2, and substrate R1′, [M_11′_]:[X_11′_]:[E1]:[M_12′_]:[X_21′_]:[E2]:[R1′] = 1:1:1:1:1:1:2. (**c**) Histogram representation of F1. The sampling interval is 7 min, 200 cycles.

In the calculation of the multiplicative weighted sum of the 2 × 2 Boolean matrix, the element in the i-th row, j-th column of the matrix F is equal to the product of the element in the i-th row of the matrix M and the corresponding element in the j-th column of the matrix X and then multiplied by the sum of their respective weights. As shown in Figure 6, the elements of the two matrices represent input signals, DNAzyme E1 and DNAzyme E2 represent weights, and the fluorescent substrate strands R1′ (5′-end modified fluorescent group FAM, 3′-end modified quencher BHQ1) and R2′ (5′-end modified fluorescent group ROX, 3′-end modified quencher BHQ2) are used to detect the output signal. In order to perform quantitative calculations, we lengthened stem S3 in the four-pronged complex to 14 bp and shortened the length of stem S6 to 9 bp. The verification process is shown in Figures S13 and S14. In this experiment, we fixed the weight to 1, and the output signal depended on the input of the matrix elements. Figure 7a is a schematic diagram of the weighted sum of the elements F1 in the first row of the output matrix F. The input auxiliary strand combination is multiplied by its respective weights to form a structure of the DNAzyme binding to double auxiliary strands, and then the same substrate is cleaved to release a fluorescent signal.

We used quantitative fluorescence experiments to verify the rationality of the F1 weighted sum. As shown in Figure 7b, when only the combination M_11′_X_11′_ is input, the combination is multiplied by the weight E1, and the substrate R1′ is digested to generate a fluorescent signal and reach the appropriate level. When only the combination M_12′_X_21′_ is input, the combination is multiplied by the weight E2, and the substrate R1′ is also digested to generate a fluorescent signal and reach the appropriate level. When the two combinations M_11′_X_11′_ and M_12′_X_21′_ are both input, the combination M_11′_X_11′_ is multiplied by the weight E1, and the combination M_12′_X_21′_ is multiplied by the weight E2 to form structures of the DNAzyme binding to double auxiliary strands. At the same time, the substrate R1′ is digested to produce a fluorescence signal, and the fluorescence value reaches a higher level; thus, the process of summing is realized. Figure 7c shows a histogram for calculating the weighted sum of F1. From the histogram, it can be intuited that when both sets of inputs are added, the fluorescence value reaches the maximum. This verifies the rationale of the weighted sum module. The summation diagram of the element F2 in the second row of the resulting matrix F is given in the Supplementary Materials (Figure S12). The above experiments prove that our mechanism of the DNAzyme binding to double auxiliary strands to cleave the substrate is feasible in the matrix multiplication weighted sum.

## 4. Conclusions

In this study, a matrix multiplication operation method was constructed using the mechanism of the DNAzyme binding to double auxiliary strands to cleave the substrate, and a weighted sum function was realized. First, we designed a mechanism of the DNAzyme binding to a single auxiliary strand to cleave the substrate. This mechanism only requires the assistance of one auxiliary strand, and E6 DNAzyme can cleave the substrate. The mechanism was optimized by adjusting the structure and sequence, and the best performance was obtained through comparative analysis of experimental results. Second, this study introduced a second auxiliary strand on the basis of the results with a single auxiliary strand and constructed a mechanism of the DNAzyme binding to double auxiliary strands to cleave the substrate. This mechanism requires two auxiliary strands to help E6 DNAzyme cleave the substrate. Based on the experience of the mechanism mediated by a single auxiliary strand, the performance of the mechanism was also optimized. The combination of two auxiliary strands was similar to the multiplication of the elements of two matrices. In the presence of E6 DNAzyme and the substrate, a result value of matrix multiplication was obtained. E6 DNAzyme was easy to synthesize, had good stability, and could work at room temperature. Most importantly, E6 DNAzyme showed a specific recognition ability, which improved the accuracy of the calculation for the matrix computing method.

In terms of scale, our scheme is theoretically expandable. By adding elements with identical structures, the order of the matrix can be increased. Therefore, these matrices composed of DNA molecules can be multiplied not only by vectors but also by matrices. The elements of the Boolean matrix are only 0 and 1, so its application is limited. Therefore, to enhance the practical value of the matrix operations we designed, integers will be introduced. Analog computation is currently used as a powerful tool for numerical computation [52] that can handle calculations on integers appropriately. Therefore, analog computation based on DNA molecules can be used to expand our matrix operations. The value of the elements in the matrix can be expressed by the concentration of DNA strands. Matrix operations based on integers will provide more possibilities for the construction of large-scale computing circuits and the solution for many engineering problems. In the field of artificial intelligence, they can be used to build neural network computing systems and intelligent information processing systems.

## Data Availability

Not applicable.

## Supplementary Materials

The following are available online at https://www.mdpi.com/article/10.3390/biom11121797/s1, Figure S1: Nupack simulations for partial DNA sequences in Table S1, Figure S2: Native PAGE analysis of the length of stem S2, Figure S3: Fluorescence image of stem S2 at 5 bp, Figure S4: Native PAGE analysis of with or without buffer area, Figure S5: Fluorescence analysis diagram of the length of stem S3. Figure S6: Fluorescence analysis diagram of the length of stem S4, Figure S7: Native PAGE analysis of with or without buffer area. Lane 1, Figure S8: Fluorescence analysis diagram of the length of stems S1 and S5, Figure S9: Fluorescence analysis diagram of the length of stems S3 and S6, Figure S10: Results of 16 independent experiments, Figure S11: Schematic diagram of matrix multiplication weighted sum, Figure S12: Comparison experiment diagram of the length of stem S3 and S6, for F1, Figure S13: Comparison experiment diagram of the length of stem S3 and S6, for F2. Table S1: Sequence of the mechanism of the DNAzyme binding to a single auxiliary strand to cleave the substrate; Table S2: Sequence of the mechanism of the DNAzyme binding to double auxiliary strands to cleave the substrate; Table S3: Sequence of Matrix multiplication operation.
