# Supplementary material for: DNA Matrix Operation Based on the Mechanism of the DNAzyme Binding to Auxiliary Strands to Cleave the Substrate
Source: Biomolecules. 2021 Nov 30;11(12):1797. doi: 10.3390/biom11121797 (PMC8698824; doi:10.3390/biom11121797)
Supplement: Supplementary file 1 [file biomolecules-11-01797-s001.zip › biomolecules-1445615-supplementary.pdf]

Supplementary

Contents

1. DNA sequence 1
2.The mechanism of the DNAzyme binding to a single auxiliary strand to cleave the substrate .... Error! Bookmark not defined.
3.The mechanism of the DNAzyme binding to double auxiliary strands to cleave the substrate......8
4. DNA matrix operation...10
5. Weighted sum of Boolean matrix multiplication 11

1.DNA sequence

In order to make gel electrophoresis gel lanes more obvious, all the DNA sequences in this experiment were designed by NUPACK. At the same time, in order to make gel electrophoresis lanes more obvious, multi-thymine (T) was employed. DNA sequences are shown in Table S1-S3.

Table S1. Sequence of the mechanism of the DNAzyme binding to a single auxiliarystrand to cleave the substrate.

Strand Name	DNA Sequence (5' to 3')
DZ0	TTATGTCAGCGATACGGTACTTGTATAGTACCGTCACCCATGTCTCTTCTCTAGT
DZ1	TTATGTCAGCGATACGGTACTTGTATAGTACCGTCACCCATGTTCTCTTCTCTAGT
DZ2	TTATGTCAGCGATACGGTACTTGTATAGTACCGTCACCCATGTTCCTCTTCTCTAGT
DZ3	TTATGTCAGCGATACGGTACTTGTATAGTACCGTCACCCATGTTCACTCTTCTCTAGT
DZ4	TTATGTCAGCGATACGGTACTTGTATAGTACCGTCACCCATGTTCAGCTCTTCTCTAGT
DZ5	TTATGTCAGCGATACGGTACTTGTATAGTACCGTCACCCATGTTCAGTCTCTTCTCTAGT
BrA00	ATCATCTCATT/rA/GGACATAATTTTTTTTTTTTTTT
BrA11	ATCATCTCAT A T/rA/GGACATAATTTTTTTTTTTTTTT
BrA22	ATCATCTCAT GA T/rA/GGACATAATTTTTTTTTTTTTTT
BrA33	ATCATCTCAT TGA T/rA/GGACATAATTTTTTTTTTTTTTT
BrA44	ATCATCTCAT CTGA T/rA/GGACATAATTTTTTTTTTTTTTT
BrA55	ATCATCTCAT ACTGA T/rA/GGACATAATTTTTTTTTTTTTTT
Aux	ACTAGAGAAGAGATGAGATGAT
BrATTT	GGACATAATTTTTTTTTTTTTTT

BrA3T	ATCATCTCATAT TGA T/rA/GGACATAATTTTTTTTTTTTTTT
BrA4T	ATCATCTCATTACTGA T/rA/GGACATAATTTTTTTTTTTTTTT
Aux-z11	CTAGAGAAGAGATGAGATGAT
Aux-z10	TAGAGAAGAGATGAGATGAT
Aux-z9	AGAGAAGAGATGAGATGAT
Aux-z8	GAGAAGAGATGAGATGAT
Aux-b9	ACTAGAGAAGAGATGAGATGA
Aux-b8	ACTAGAGAAGAGATGAGATG
Aux-b7	ACTAGAGAAGAGATGAGAT
Aux-b6	ACTAGAGAAGAGATGAGA

Table S2. Sequence of the mechanism of the DNAzyme binding to double auxiliary strands to cleave the substrate

Strand Name	DNA Sequence (5' to 3')
DE3	TGTACACTCATCATCAGCGATACGGTACTTGTATAGTACCGTCACCCATGT TCACTCTTCTCTAGT
ErA3d	TACTTGATCTTGAT/rA/GGATTACTCATCTA
ErA3T	TACTTGATCTATTGAT/rA/GGATTATACTCATCTA
DB	TACTTGATCTATTGATA
Aux1-z12	ACTAGAGAAGAGAGATCAAGTA
Aux1-z11	CTAGAGAAGAGAGATCAAGTA
Aux1-z10	TAGAGAAGAGAGATCAAGTA
Aux1-z9	AGAGAAGAGAGATCAAGTA
Aux1-z8	GAGAAGAGAGATCAAGTA
Aux2-z12	TAGATGAGTAGATGAGTGTACA
Aux2-z11	TAGATGAGTAGATGAGTGTAC
Aux2-z10	TAGATGAGTAGATGAGTGTA
Aux2-z9	TAGATGAGTAGATGAGTGT
Aux2-z8	TAGATGAGTAGATGAGTG
Aux1-d10	CTAGAGAAGAGAGATCAAGTA
Aux1-d9	CTAGAGAAGAGAGATCAAGT
Aux1-d8	CTAGAGAAGAGAGATCAAG
Aux1-d7	CTAGAGAAGAGAGATCAA
Aux1-d6	CTAGAGAAGAGAGATCA
Aux2-d10	TAGATGAGTAGATGAGTGTAC
Aux2-d9	AGATGAGTAGATGAGTGTAC
Aux2-d8	GATGAGTAGATGAGTGTAC
Aux2-d7	ATGAGTAGATGAGTGTAC
Aux2-d6	TGAGTAGATGAGTGTAC

Table S3. Sequence of Matrix multiplication operation

Strand Name	DNA Sequence (5' to 3')
M_{11}	GCAGCTTGCAGATGAGTGTACA
M_{12}	GCAGCTTGCAGTGTTGATCTGT
M_{21}	TGCTGTAGCAGATGAGTGTACA
M_{22}	TGCTGTAGCAGTGTTGATCTGT
X_{11}	ACTAGAGAAGAGAGATCAAGAT
X_{21}	TACGAGTGATAGAGATCAAGAT
E1 (DE3)	TGTACACTCATCATCAGCGATACGGTACTTGTATAGTACCGTCACCCATGTTCACTCTTCTCTAGT
E2	ACAGATCAACACATCAGCGATACGGTACTTGTATAGTACCGTCACCCATGTTCACTATCACTCGTA
R1	/FAM/—ATCTTGATCTATTGAT/rA/GGATTATGCAAGCTGC—/BHQ1/
R2	/ROX/—ATCTTGATCTATTGAT/rA/GGATTATGCTACAGCA—/BHQ2/
M_{11}'	ATGCAGACTTATGCGATGAGTGTACA
M_{12}'	ATGCAGACTTATGCGTGTTGATCTGT
M_{21}'	TCAGTGATGATAGCGATGAGTGTACA
M_{22}'	TCAGTGATGATAGCGTGTTGATCTGT
X_{11}'	ACTAGAGAAGAGAGATCAAGA
X_{21}'	TACGAGTGATAGAGATCAAGA
R1'	/FAM/—ATCTTGATCTATTGAT/rA/GGATTAGCATAAGTCTGCAT—/BHQ1/
R2'	/ROX/—ATCTTGATCTATTGAT/rA/GGATTAGCTATCATCACTGA—/BHQ2/
M_{11}'-13	TGCAGACTTATGCGATGAGTGTACA
M_{12}'-13	TGCAGACTTATGCGTGTTGATCTGT
M_{21}'-13	CAGTGATGATAGCGATGAGTGTACA
M_{22}'-13	CAGTGATGATAGCGTGTTGATCTGT
X_{11}'-10	ACTAGAGAAGAGAGATCAAGAT
X_{21}'-10	TACGAGTGATAGAGATCAAGAT

All of the sequences used in this work were designed using Nupack.

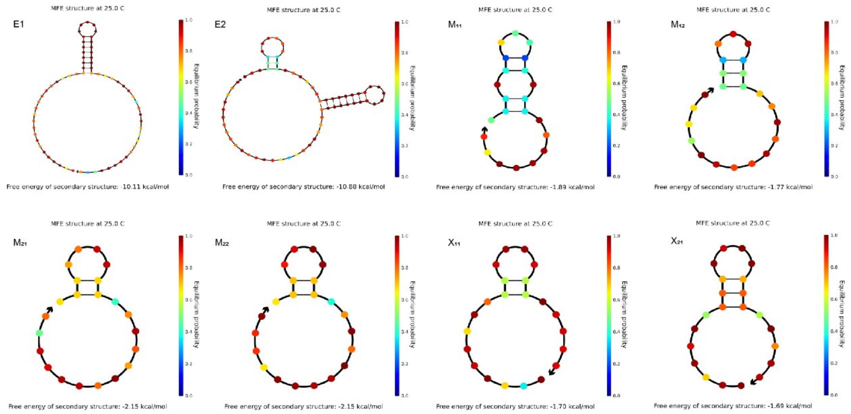

Figure S1. Nupack simulations for partial DNA sequences in table S1.

2. The mechanism of the DNAzyme binding to a single auxiliary strand to cleave the substrate

To explore the effect of the length of stem S2 on the mechanism of the DNAzyme binding to a single auxiliary strand to cleave the substrate, we conducted a gradient test of the length of stem S2. The gel electrophoresis experiment with the length of stem S2 from 0 bp to 5 bp is shown in Figure.S2. The results showed that when the stem S2 was 3 bp, 4 bp, 5 bp, the auxiliary strand was added and DNAzyme began to cleave the substrate.

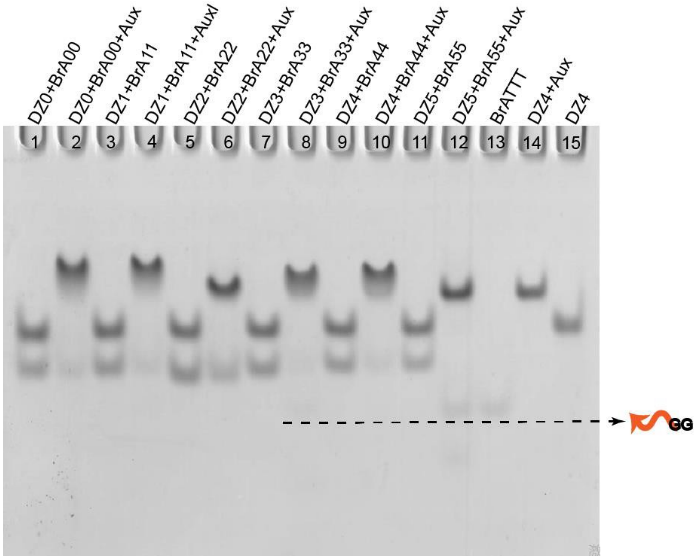

Figure S2. Native PAGE analysis of the length of stem S2. The strands and complexes involved are marked above the lane number. Lane 1, DNAzyme DZ0 and substrate strand BrA00; lane 2, adding the input strand Aux to the mixed solution of DNAzyme DZ0 and the substrate strand BrA00. lane3, DNAzyme DZ1 and substrate strand BrA11; lane 4, adding the input strand Aux to the mixed solution of DNAzyme DZ1 and the substrate strand BrA11. lane 5, DNAzyme DZ2 and substrate strand BrA22; lane 6, adding the input strand Aux to the mixed solution of DNAzyme DZ2 and the substrate strand BrA22. lane 7, DNAzyme DZ3 and substrate strand BrA33; lane 8, adding the input strand Aux to the mixed solution of DNAzyme DZ3 and the substrate strand BrA33. lane 9, DNAzyme DZ4 and substrate strand BrA44; lane 10, adding the input strand Aux to the mixed solution of DNAzyme DZ4 and the substrate strand BrA44. lane 11, DNAzyme DZ5 and substrate strand BrA55; lane 12, adding the input strand Aux to the mixed solution of DNAzyme DZ5 and the substrate strand BrA55. lane 13, product strand BrATTT; lane 14, DNAzyme DZ4 and input strand Aux complex; lane15, DNAzyme DZ4.

In the mechanism of the DNAzyme binding to a single auxiliary strand to cleave the substrate, when the stem S2 is 5 bp and no input is added, and the DNAzyme slowly cleaves the substrate, which causes the fluorescence signal to rise and leakage problems occur. Fluorescence analysis is shown in Figure.S3.

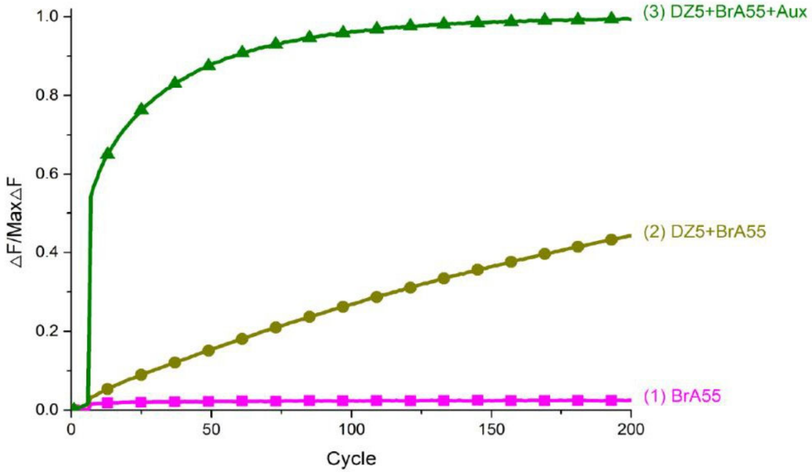

Figure S3. Fluorescence image of stem S2 at 5 bp. Curve (1), only fluorescent substrate strand BrA55 in solution; curve (2), there is DNAzyme DZ5 and fluorescent substrate strand BrA55 in the solution; curve (3), adding the input strand Aux to the mixed solution of DNAzyme DZ5 and the fluorescentsubstrate strand BrA55. Sampling interval is 4 minutes, 200 cycles.

To optimize the experiment and make the mechanism of the DNAzyme binding to a single auxiliary strand to cleave the substrate achieve a better cutting effect, we added a buffer area between the stem S2 and S4, and did a comparison test with or without buffer area. The result of gel electrophoresis is shown in Figure.S4. The results showed that with the addition of buffer area, the mechanism of enzyme digestion effect is better.

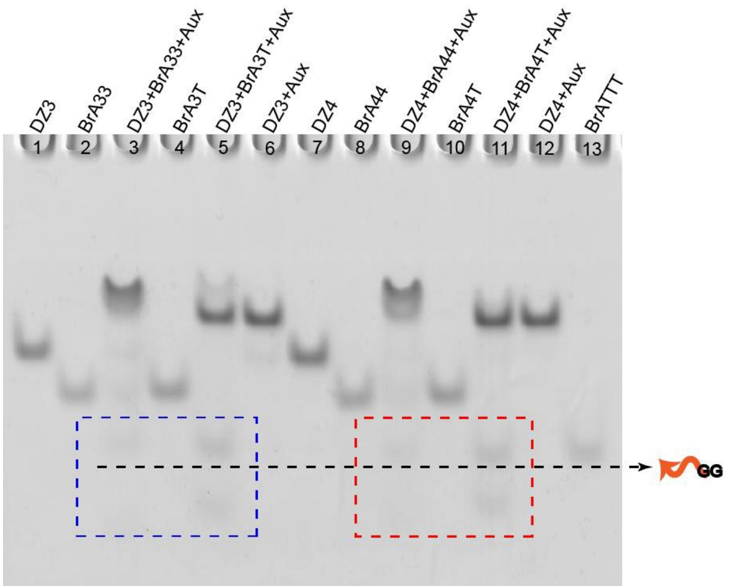

Figure S4. Native PAGE analysis of with or without buffer area. The strands and complexes involved are marked above the lane number. Lane 1, DNAzyme DZ3; lane 2, substrate strand BrA33; lane 3,adding the input strand Aux to the mixed solution of DNAzyme DZ3 and the substrate strand BrA33,there is no buffer area at this time; lane 4, substrate strand BrA3T; lane 5, adding the input strand Aux to the mixed solution of DNAzyme DZ3 and the substrate strand BrA3T, there is a buffer at this time; lane 6, DNAzyme DZ3 and input strand Aux complex; lane 7, DNAzyme DZ4; lane 8, substrate strand BrA44; lane 9, adding the input strand Aux to the mixed solution of DNAzyme DZ4 and the substrate strand BrA44, there is no buffer area at this time; lane 10, substrate strand BrA4T; lane 11, adding the input strand Aux to the mixed solution of DNAzyme DZ4 and the substrate strand BrA4T, there is a buffer at this time; lane 12, DNAzyme DZ4 and input strand Auxcomplex; lane 13, product strand BrATTT.

To optimize the experiment, a gradient experiment was performed on the length of the stem S3. The fluorescence analysis diagram of the length of stem S3 from 8 bp to 12 bp is shown in Figure.S5. The results showed that when the length of stem S3 was 11 bp and 12 bp, the fluorescence increased the most.

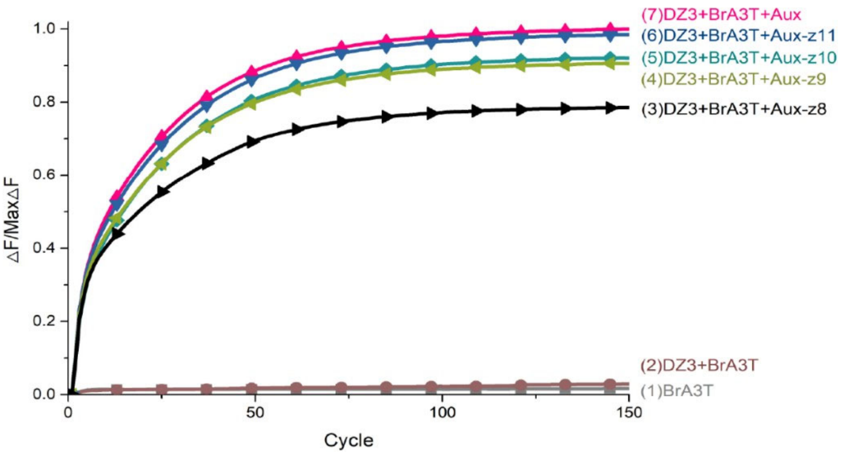

Figure S5. Fluorescence analysis diagram of the length of stem S3. Curve (1), only the fluorescent substrate strand BrA3T; curve (2), only DNAzyme DZ3 and fluorescent substrate strand BrA3T; curve (3), adding the input strand Aux-z8 to the mixed solution of DNAzyme DZ3 and the fluorescent substrate strand BrA3T, at this time, the stem S3 is 8 bp; curve (4), adding the input strand Aux-z9 to the mixed solution of DNAzyme DZ3 and the fluorescent substrate strand BrA3T, at this time, the stem S3 is 9 bp ; curve (5), adding the input strand Aux-z10 to the mixed solution of DNAzyme DZ3 and the fluorescent substrate strand BrA3T, at this time, the stem S3 is 10 bp ; curve (6), adding the input strand Aux-z11 to the mixed solution of DNAzyme DZ3 and the fluorescent substrate strand BrA3T, at this time, the stem S3 is 11 bp; curve (7), adding the input strand Aux-z12 to the mixed solution of DNAzyme DZ3 and the fluorescent substrate strand BrA3T, at this time, the stem S3 is 12 bp;.

After optimizing the length of stem S3, a gradient test was carried out on the length of stem S4. The fluorescence analysis diagram of the length of stem S4 from 6 bp to 10 bp is shown in Figure.S6. The results showed that when the length of stem S4 is 10 bp, the fluorescence rises the most.

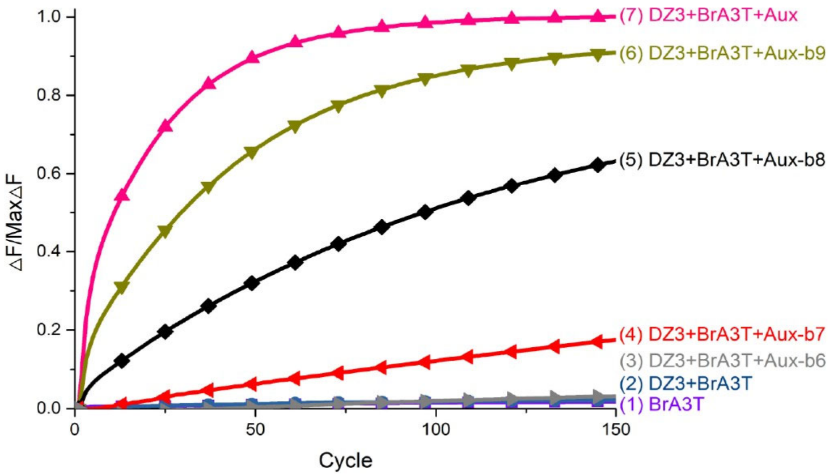

Figure S6. Fluorescence analysis diagram of the length of stem S4. Curve (1), only the fluorescent substrate strand BrA3T; curve (2), only DNAzyme DZ3 and fluorescent substrate strand BrA3T; curve (3), adding the input strand Aux-b6 to the mixed solution of DNAzyme DZ3 and the fluorescent substrate strand BrA3T, at this time, the stem S4 is 6bp; curve (4), adding the input strand Aux-b7 to the mixed solution of DNAzyme DZ3 and the fluorescent substrate strand BrA3T, at this time, the stem S4 is 7bp ; curve (5), adding the input strand Aux-b8 to the mixed solution of

DNAzyme DZ3 and the fluorescent substrate strand BrA3T, at this time, the stem S4 is 8bp ; curve (6), adding the input strand Aux-b9 to the mixed solution of DNAzyme DZ3 and the fluorescent substrate strand BrA3T, at this time, the stem S4 is 9bp; curve (7), adding the input strand Aux-b10 to the mixed solution of DNAzyme DZ3 and the fluorescent substrate strand BrA3T, at this time, the stem S4 is 10bp;

3. The mechanism of the DNAzyme binding to double auxiliary strands to cleave the substrate

To optimize the mechanism of the DNAzyme binding to double auxiliary strands to cleave the substrate achieve a better cutting effect, we added buffer area between the stem S2 and S3, S4 and S6, did a comparison test with or without buffer area. The result of gel electrophoresis is shown in Figure.S7. The results showed that with the addition of buffer area, the mechanism of enzyme digestion effect is better.

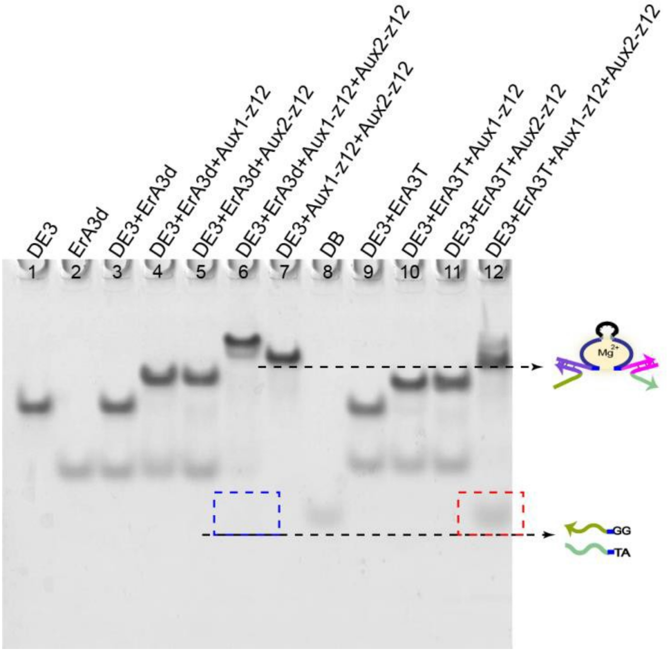

Figure S7. Native PAGE analysis of with or without buffer area. Lane 1, DNAzyme DE3; lane 2, substrate strand ErA3d; lane 3, DNAzyme DE3 and substrate strand ErA3d; lane 4,adding only one auxiliary strand Aux1-z12 to the mixed solution of DNAzyme DE3 and the substrate strand ErA3d,there is no buffer area at this time; lane 5, adding another auxiliary strand Aux2-z12 to the mixed solution of DNAzyme DE3 and the substrate strand ErA3d, there is no buffer area at this time; lane 6, both auxiliary strands are added to mixed solution of DNAzyme DE3 and the substrate strand ErA3d, there is no buffer area at this time; lane 7, complex of DNAzyme and two auxiliary strands; lane 8, product strand comparison; lane 9, DNAzyme DE3 and substrate strand ErA3T; lane 10, adding only one auxiliary strand Aux1-z12 to the mixed solution of DNAzyme DE3 and the substrate strand ErA3T, add buffer area at this time; lane 11, adding another auxiliary strand Aux2- z12 to the mixed solution of DNAzyme DE3 and the substrate strand ErA3T, add buffer area at thistime; lane 12, both auxiliary strands are added to mixed solution of DNAzyme DE3 and the substrate strand ErA3T, add buffer area at this time.

Optimized the mechanism of the DNAzyme binding to auxiliary double strands to cleave the substrate, and conducted gradient experiments on the length of stems S1 and S5 from 8 bp to 12 bp, The fluorescence analysis is shown in Figure.S8, and the results showed that when the length of stems S1 and S5 is 11 bp, the DNAzyme cutting effect is the best.

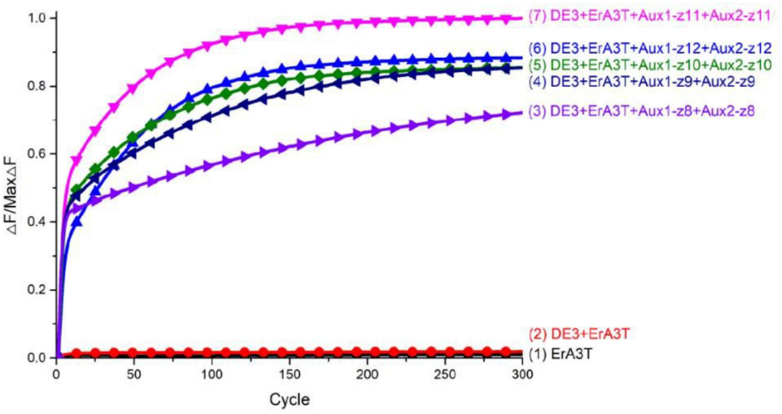

Figure S8. Fluorescence analysis diagram of the length of stems S1 and S5. Curve (1), only the fluorescent substrate strand ErA3T; curve (2), only DNAzyme DE3 and fluorescent substrate strand ErA3T; curve (3), adding the input strands Aux1-z8 and Aux2-z8 to the mixed solution of DNAzyme DE3 and the fluorescent substrate strand ErA3T, at this time, the stems S1 and S5 is 8 bp; curve (4), adding the input strands Aux1-z9 and Aux2-z9 to the mixed solution of DNAzyme DE3 and the fluorescent substrate strand ErA3T, at this time, the stems S1 and S5 is 9 bp; curve (5), adding the input strands Aux1-z10 and Aux2-z10 to the mixed solution of DNAzyme DE3 and the fluorescent substrate strand ErA3T, at this time, the stems S1 and S5 is 10 bp; curve (6), adding the input strands Aux1-z12 and Aux2-z12 to the mixed solution of DNAzyme DE3 and the fluorescent substrate strand ErA3T, at this time, the stems S1 and S5 is 12 bp; curve (7), adding the input strands Aux1-z11 and Aux2-z11 to the mixed solution of DNAzyme DE3 and the fluorescent substrate strand ErA3T, at this time, the stems S1 and S5 is 11 bp.

After optimizing the length of stems S1 and S5, gradient experiments was carried out on the length of stems S3 and S6. The fluorescence analysis diagram of the length of stems S3 and S6 from 6 bp to 10 bp is shown in Figure.S9. The results showed that when the length of stems S3 and S6 is 10 bp, the fluorescence rises the most.

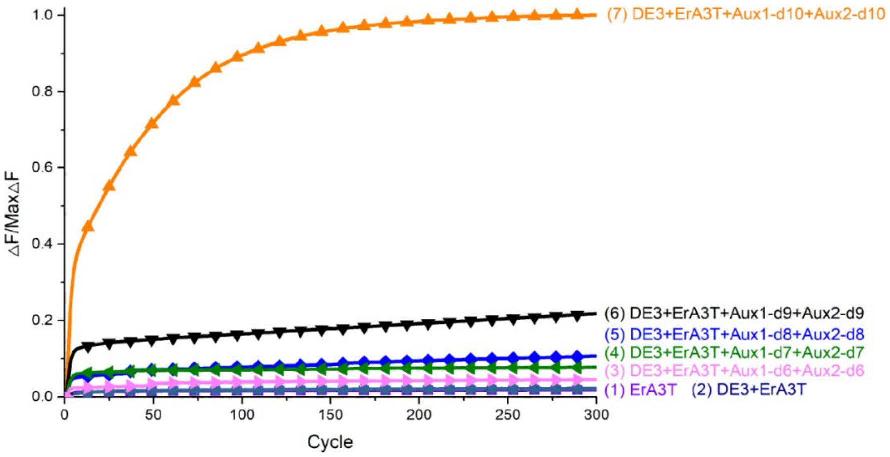

Figure S9. Fluorescence analysis diagram of the length of stems S3 and S6. Curve (1), only the fluorescent substrate strand ErA3T; curve (2), only DNAzyme DE3 and fluorescent substrate strand ErA3T; curve (3), adding the input strands Aux1-d6 and Aux2-d6 to the mixed solution of DNAzyme DE3 and the fluorescent substrate strand ErA3T, at this time, the stems S3 and S6 is 6 bp; curve (4), adding the input strands Aux1-d7 and Aux2-d7 to the mixed solution of DNAzyme DE3 and the fluorescent substrate strand ErA3T, at this time, the stems S3 and S6 is 7 bp; curve (5), adding the input strands Aux1-d8 and Aux2-d8 to the mixed solution of DNAzyme DE3 and the fluorescent substrate strand ErA3T, at this time, the stems S3 and S6 is 8 bp; curve (6), adding the input strands Aux1-d9 and Aux2-d9 to the mixed solution of DNAzyme DE3 and the fluorescent substrate strand ErA3T, at this time, the stems S3 and S6 is 9 bp; curve (7), adding the input strands Aux1-d10 and Aux2-d10 to the mixed solution of DNAzyme DE3 and the fluorescent substrate strand ErA3T, at this time, the stems S3 and S6 is 10 bp.

4. DNA matrix operation

The other 16 independent experiments of DNA matrix multiplication are shown in Figure.S10. The 16 independent experiments correspond to 4 different M matrices multiplied by 4 different X matrices. The blue curve represents the value of F1, the red curve represents the value of F2.

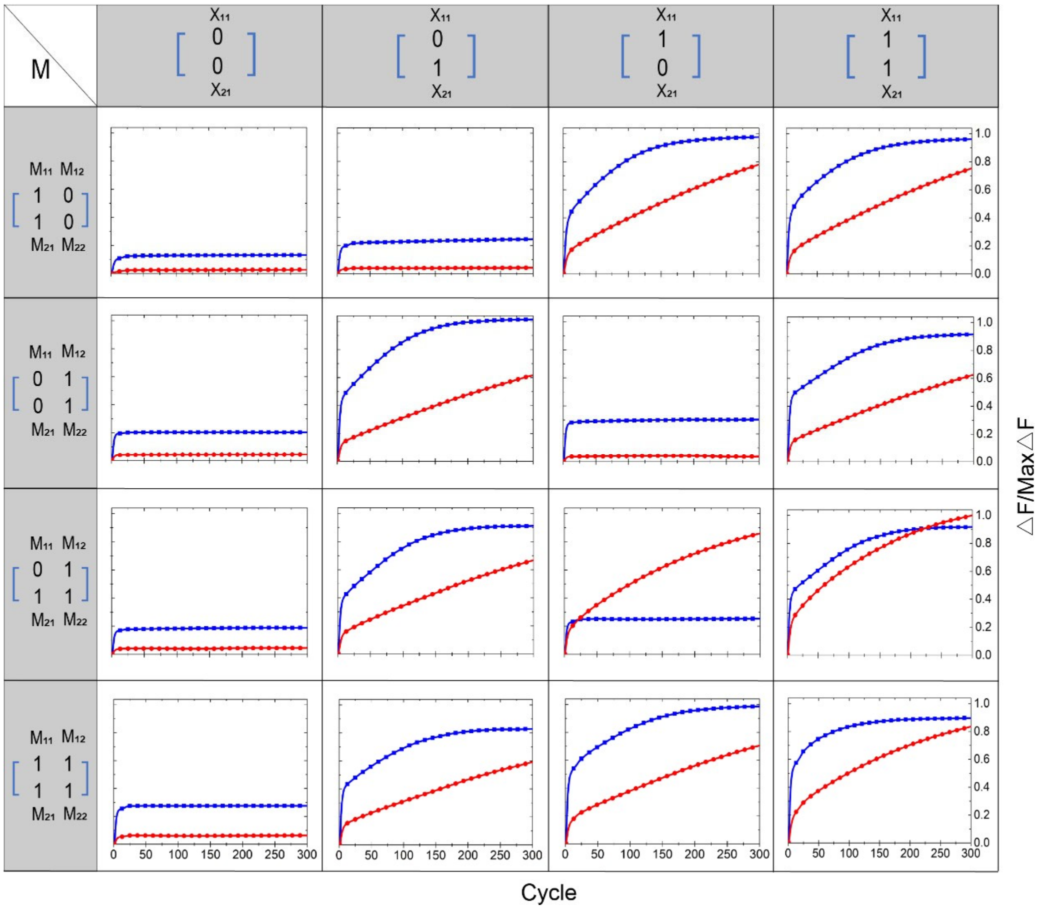

Figure S10. Results of 16 independent experiments. Before adding the element strands of the two matrices, first mix the two DNAzymes and the two substrate strands of the reporting module. The sampling interval is 4 minutes and 300 cycles. All data represent the average of three replicates.

5. Weighted sum of Boolean matrix multiplication

Figure.S11 a is a schematic diagram of the summation of the element F2 in the second row of the result matrix F. The fluorescence quantitative experiment verified the rationality of calculating the weighted sum of F2, as shown in Figure.S11 b. Similar to F1, when only the combination M_{21}'X_{11}' is input, the combination is multiplied by the weight E1, and the substrate R2' is digested to generate a fluorescent signal and reach a level; When only the combination M_{22}'X_{21}' is input, the combination is multiplied by the weight E2, which also cuts the substrate R2', produces a fluorescent signal and reaches a level; When the two combinations M_{21}'X_{11}' and M_{22}'X_{21}' are both input, the combination M_{21}'X_{11}' is multiplied by the weight E1, and the combination M_{22}'X_{21}' multiply by the weight E2, form structures of DNAzyme binding to double auxiliary strands and simultaneously digest the substrate R2' to generate the same fluorescence signal, the fluorescence value reaches a higher level, and the process of summation is realized. Figure.S11 c is a histogram for calculating the weighted sum of F2. From the histogram, it is more intuitive to show that when both sets of inputs are added, the fluorescence value reaches the maximum. This verifies the rationality of the weighted sum module.

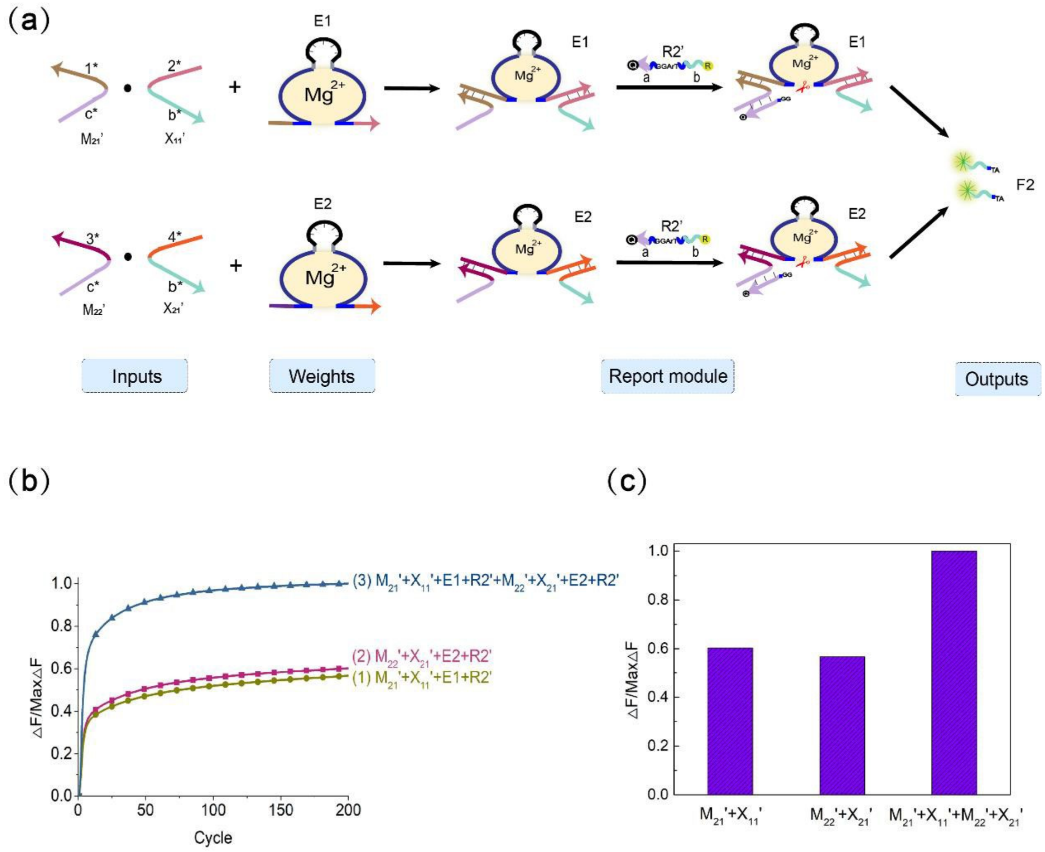

Figure S11. Schematic diagram of matrix multiplication weighted sum. (a) Schematic diagram of the weighted sum process of the first-row value F2 of the output matrix F. (b) Normalized fluorescence image of F2. Curve (1), combination of M_{21}'X_{11}' and weight E1, substrate R2', [M_{21}']:[X_{11}']: [E1] : [R2']=1:1:1:2; curve (2), combination of M_{22}'X_{21}' and weight E2, substrate R2',

[M_{22}']:[X_{21}']: [E2]: [R2']=1:1:1:2; curve (3), Combinations M21'X11', M22'X21', weights E1, E2 and substrate R2', [M_{21}']:[X_{11}']:[E1]:[M_{22}']:[X_{21}']:[E2]:[R2']=1:1:1:1:1:1:2. (c) Histogram representation of F2. The sampling interval is 7 minutes, 200 cycles.

In order to quantitatively calculate the weighted sum, the combined digestion structures mediated by the double auxiliary strands need to be disposable consumable. The method is to lengthen the stem S3 to 13 bp and 14 bp, so that the substrate does not fall after being cleaved. The results of F1 are shown in Figure S12 (b) and (d). When only one input exists, the fluorescence value does not reach saturation, has been showing an upward trend, and gradually tends to the situation when two inputs are added at the same time. Considering that the strand replacement may occur due to the

binding of stem S6 too stable, therefore, the stem S6 was shortened to 9 bp. As shown in Figure S12(a) and (c), when the stem S3 is 13 bp, the effect is still unsatisfactory; when the stem S3 is 14 bp, the single-input fluorescence value is about half of the double-input fluorescence value, and the result is ideal. Therefore, through comparative experiments, it is determined that the length of stem S3 is 14 bp and the length of stem S6 is 9 bp.

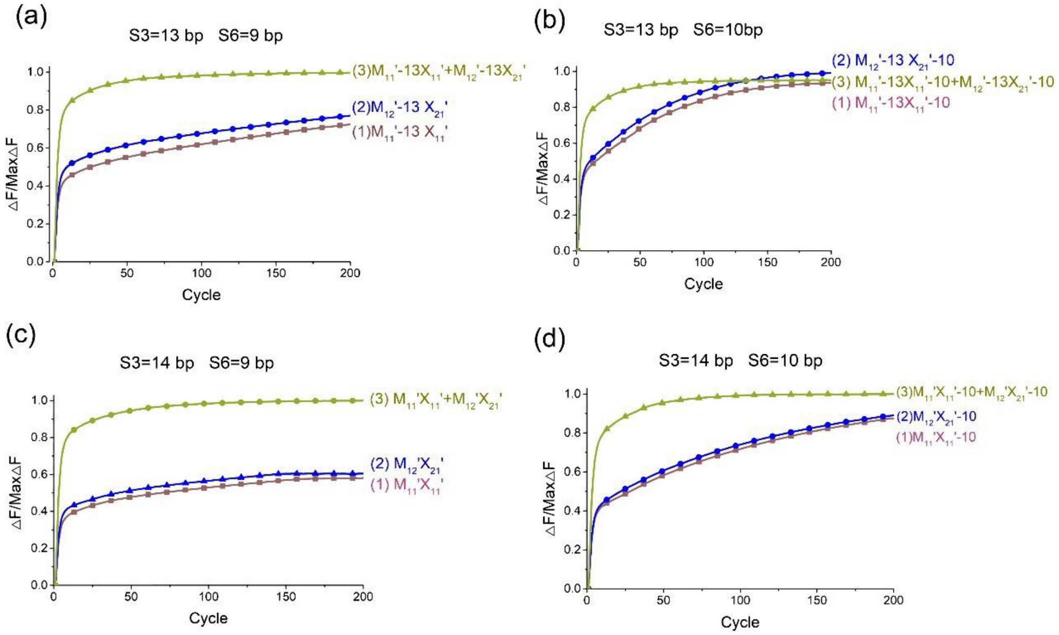

Figure. S12 Comparison experiment diagram of the length of stem S3 and S6, for F1. In the four illustrations, the reaction represented by curve (1) has been added with the same substrate E1 and R1'; the reaction represented by curve (2) has been added with E2 and R1'; the reaction represented by curve (3) has been added with E1, E2 and R1'. (a) Curve (1), $[M_{11}'-13]$:$[X_{11}']$: [E1]:[R1']=1:1:1:2, $[M_{11}'-13]$=0.3 μM. curve (2), $[M_{12}'-13]$:$[X_{21}']$:[E2]:[R1']=1:1:1:2, $[M_{12}'-13]$=0.3 μM. curve (3), $[M_{11}'-13]$:$[X_{11}']$:[E1]:$[M_{12}'-13]$:$[X_{21}']$:[E2]:[R1']=1:1:1:1:1:1:2. Illustrations (b), (c) and (d) have the same input and substrate concentration ratio for each curve. The sampling interval is 7 minutes, 200 cycles.

The verification process of value F2 is the same as that of value F1, the experimental results are shown in Figure S13, when the stem S3 is 14 bp and the stem S6 is 9 bp, the effect is the best.

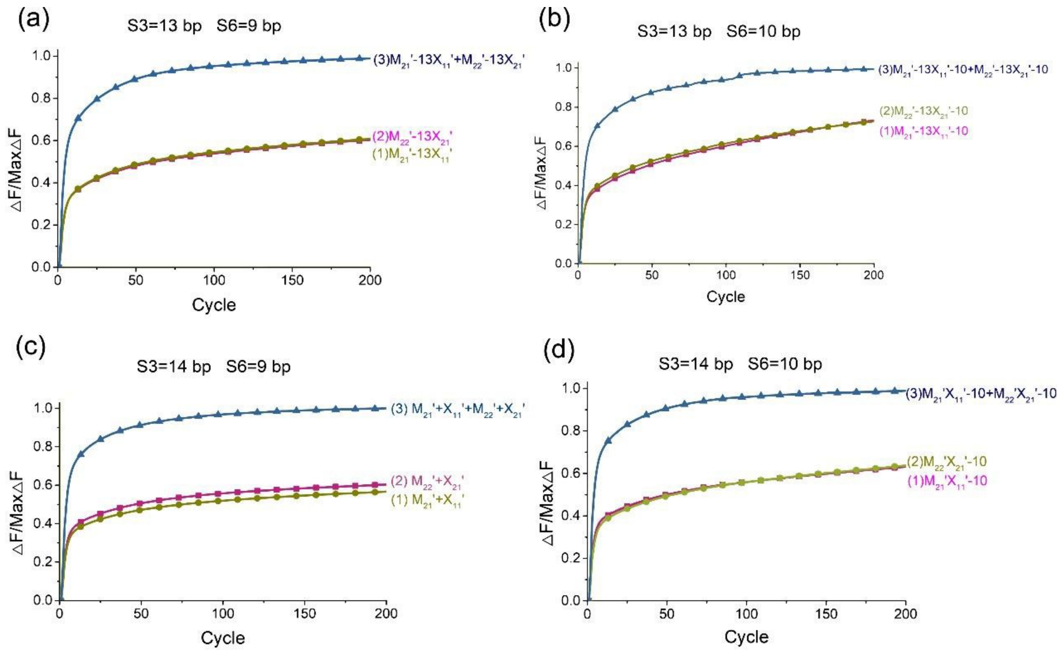

Figure. S13 Comparison experiment diagram of the length of stem S3 and S6, for F2. In the four illustrations, the reaction represented by curve (1) has been added with the same substrate E1 and R2'; the reaction represented by curve (2) has been added with E2 and R2'; the reaction represented by curve (3) has been added with E1, E2 and R2'. (a) Curve (1), [M_{21}'-13]:[X_{11}']: [E1]:[R2']=1:1:1:2, [M_{21}'-13]=0.3 μM. curve (2), [M_{22}'-13]:[X_{21}']:[E2]:[R2']=1:1:1:2, [M_{22}'-13]=0.3 μM. curve (3), [M_{21}'-13]:[X_{11}']:[E1]:[M_{22}'-13]:[X_{21}']:[E2]:[R2']=1:1:1:1:1:1:2. Illustrations (b), (c) and (d) have the same input and substrate concentration ratio for each curve. The sampling interval is 7 minutes, 200 cycles.
